# Unilateral Exudative Retinal Detachment as the Sole Presentation of Relapsing Acute Lymphoblastic Leukemia

**DOI:** 10.5505/tjh.2012.72623

**Published:** 2012-06-15

**Authors:** Fatih Mehmet Azık, Arsen Akıncı, Tülin Revide Şaylı, Vildan Kosan Çulha, Kuddusi Teberik, Mehmet Yasin Teke, Fatih Gürbüz

**Affiliations:** 1 Diskapi Children’s Hospital, Department of Pediatric Hematology, Ankara, Turkey; 2 Ulucanlar Eye Hospital, Ankara, Turkey

**Keywords:** Acute lymphoblastic leukemia, Exudative retinal detachment, Relapse

## Abstract

Ocular findings are rarely the initial symptom of leukemia, although up to 90% of all leukemia patients have fundus changes during the course of the disease. Herein we report a relapsing acute lymphoblastic leukemia patient with thesole presentation of sudden visual loss and exudative retinal detachment. An 8-year-old boy with acute lymphoblasticleukemia developed sudden visual loss during his first remission period. Bullous retinal detachment with total afferentpupillary defect was observed. Orbital magnetic resonance imaging revealed an intraocular mass lesion; simultaneouslyobtained bone marrow and cerebrospinal fluid samples showed no evidence of leukemic cells. Following local irradiation,and systemic and intrathecal chemotherapy the mass disappeared. Local irradiation, and systemic and intrathecalchemotherapy effectively controlled the isolated ocular relapse of acute lymphoblastic leukemia and eliminated the needfor enucleation.

## Abstract (tr)

Löseminin seyrinde fundus değişiklikleri tüm hastaların %90’ında görülmesine rağmen, lösemide göz tutulumu nadiren ilk bulgu olarak karşımıza çıkar. Burada, ani görme kaybı ve eksudatif retina dekolmanının akut lenfoblastik lösemili bir olguda relapsın ilk bulgusu olarak saptandığını bildiriyoruz. Sekiz yaşında akut lenfoblastik lösemi tanısı alan bir erkek çocukta birinci remisyon döneminde ani görme kaybı gelişti. Total afferent pupiller defekt ile birlikte büllöz retina dekolmanı tespit edildi. Orbital manyetik rezonans görüntüleme ile intraokuler kitle lezyonu olduğu belirlendi. Eş zamanlı yapılan kemik iliği ve beyin omurilik sıvısı incelemelerinde lösemik tutulum saptanmadı. Lokal radyoterapi sonrası sistemik ve intratekal kemoterapi uygulandı, kitlede rezolusyon gelişti. Sonuç olarak, akut lenfoblastik lösemide görülebilen izole göz relapsının kontrol altına alınmasında lokal radyoterapi, sistemik ve intratekal kemoterapinin etkili olduğunu ve enükleasyon gereksinimini ortadan kaldırdığını belirtiyoruz.

## INTRODUCTION

Ocular symptoms in leukemia patients may be due to the direct effect of leukemic cells on ocular tissues or secondary to such disease-related complications as anemia, thrombocytopenia, and hyperviscosity syndrome [1]. Acute leukemias are associated with eye involvement more commonly than chronic leukemias [2]. The choroid and retina are the most common sites of leukemic involvement [3], and leukemic involvement in the iris, ciliary body, optic nerve, and conjunctiva, as well as leukemic hypopyon have also been reported [4,5]. Herein we report a relapsing acute lymphoblastic leukemia (ALL) patient with unilateral sudden visual loss and exudative retinal detachment as the sole presentation, as well as a discussion of the outcome following local irradiation, and systemic and intrathecal chemotherapy.

## CASE

An 8-year-old boy diagnosed as pre-B ALL [flow cytometry; CD 10 (%92), CD 19 (%96), CD 20 (%86), CD22 (%80)] was treated according to the St. Jude Total XIII high-risk (HR) protocol. Cerebral spinal fluid (CSF) was normal. According to the treatment protocol, due to the risk of central nervous system relapse radiotherapy (RT) was not administered. During the 97th week of treatment (27 months after the diagnosis) the patient developed unilateral sudden visual loss during his first remission period. 

**Figure 1:**
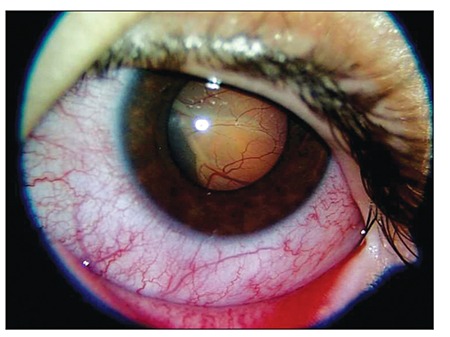
Slit-lamp photograph of the left eye at presentationshows exudative retinal detachment.

His best-corrected visual acuity was 10/10 in the right eye, whereas light perception was absent in the left eye. Color vision could not be evaluated in the left eye and was 12/12 in the right eye. There was total afferent pupillary defect in the left eye. Eye movements were full. Intraocular pressure was within normal limits in both eyes. Slit-lamp examination showed bullous exudative retinal detachment in the left eye and a completely normal right eye (Figure 1). 

**Figure 2:**
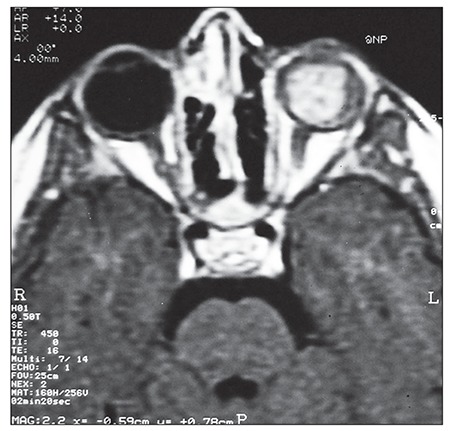
Orbital MRI shows a 2 x 1.5-cm mass lesion with irregular borders completely filling the bulbus oculi.

Ocular ultrasonographic examination showed total retinal detachment and a mass lesion that completely filled the bulbus oculi. Hyperechoic lesions indicative of calcification were not noted. Orbital magnetic resonance imaging (MRI) showed a 2 x 1.5-cm mass lesion with irregular borders that completely filled the bulbus oculi (Figure 2). Bone marrow and CSF examinations were normal. The mass in the bulbus oculi was considered as ocular relapse, though it was not biopsy proven. 

**Figure 3:**
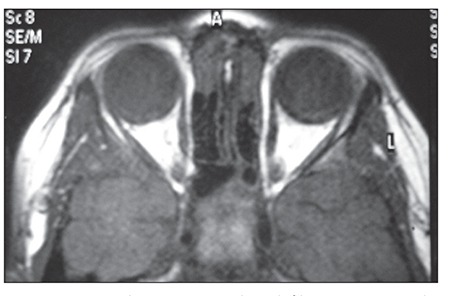
MRI shows crescent-shaped fibrotic tissue in theposterior region of the left bulbus oculi, which was considered calcification without evidence of a mass.

As soon as ocular relapse was diagnosed, re-induction therapy was initiated according to the St. Jude Total XIII-HR protocol [6]. The patient received orbital RT at a dose of 2340 cGy in 10 fractions. Follow-up orbital MRI showed crescent-shaped fibrotic tissue at the posterior region of the left bulbus oculi, which was considered calcification, but there was no evidence of a mass related with ocular relapse (Figure 3). Although his visual capacity didn`t recover, the patient remained in remission for 18 months following ocular relapse. Extramedullary relapse treatment was continued, according to the St. Jude Total XIII-HR protocol. I had written informed consent and there is no conflict of interest related to manuscript.

## DISCUSSION

Ocular involvement in children with ALL, which is typically bilateral, is usually observed with concomitant central nervous system and/or bone marrow relapse [7,8]. Improved leukemia therapies for children have resulted in prolonged remission; even with extramedullary relapses in the most common ones (bone marrow, testes, brain, and spinal cord). Ocular symptoms of ALL may be more striking than systemic symptoms, and may be the primary reason for presentation at the time of initial diagnosis. Nonetheless, exudative retinal detachment as the sole presentation of relapsing ALL in the absence of concomitant central nervous system and/or bone marrow relapse is unusual, and only 3 such cases have been reported [9,10,11]. 

The differential diagnosis in leukemic patients with exudative retinal detachment includes central serous choroidopathy, uveal effusion syndrome, choroidal neovascularization, Harada’s syndrome, congenital optic disc pits, choroidal melanoma, and severe intraocular hypotony [12]. All of the possibilities which were mentioned above, excluded in the present case due to age, patient history and ophthalmologic examination findings. 

Primack et al. reported a 3.5-year-old boy with a history of ALL that presented with total retinal detachment during a period of remission. Painful glaucoma that was unresponsive to medical therapy necessitated enucleation. Histopathologic examination showed leukemic infiltration of the retina and optic nerve. The child remained leukemia-free for 3 years following systemic and intrathecal chemotherapy, and craniospinal RT. This was the first reported case of relapsing ALL with retinal detachment as the sole presentation [11].

Schmiegelow et al. reported a 17-year-old female ALL patient with serous retinal detachment and choroidal hemorrhagic infiltration as the presenting signs of relapse, without concurrent bone marrow or central nervous system involvement 1 year after cessation of therapy. No malignant cells were observed in subretinal fluid obtained during choroidal biopsy performed via sclerotomy. She was treated with prednisone, topical corticosteroid, and atropine, which resulted in normalization of vision and clinical findings; however, retinal detachment and choroidal infiltration recurred 5 months later. Pars plana vitrectomy and retinotomy were performed to remove subretinal tissue, which showed that there was leukemic infiltration in the choroid and concurrent bone marrow relapse [13]. 

The presented patient is the fourth reported case of exudative retinal detachment as the sole presenting sign of relapsing ALL in the absence of concomitant systemic involvement. We did not confirm the presence of leukemic cells in the patient’s bulbus oculi, because we did not obtain a biopsy specimen due to technical difficulties. We did observe a new growing mass in the bulbus oculi via ultrasonography and MRI, and considered the findings as leukemic relapse. As a general rule, newly observed growing mass lesions are regarded as metastasis in cancer patients unless proved otherwise. The mass in the presented patient responded to local irradiation, and systemic and intrathecal chemotherapy. The mass disappeared, leaving behind crescent-shaped fibrotic tissue in the bulbus oculi. This response confirmed the diagnosis of the mass as leukemic infiltration. There are different therapeutic options for leukemic infiltration of the bulbus oculi in ALL patients; evisceration, enucleation, local RT, and systemic chemotherapy are methods that can be combined, according to each patient (9,10,11,13,14).

In conclusion, local irradiation, and systemic and intrathecal chemotherapy effectively controlled the isolated ocular relapse of ALL in the presented case, and eliminated the necessity for enucleation. 

**Conflict of Interest Statement**

The authors of this paper have no conflicts of interest, including specific financial interests, relationships, and/or affiliations relevant to the subject matter or materials included.
